# Reduction in the Number of Daily Lectures at Medical Colleges

**Published:** 1848-10

**Authors:** 


*Reduction in the number of daily Lectures at Medical Colleges.*— In connection with the subject of the extension of the lecture term, the inquiry arises, whether, with an extended session, it is best to diminish the number of lectures given daily. On this subject there seems to be a difference of opinion. In the annual announcement of the *Jefferson Medical College*, of Pennsylvania, we find the following remarks indicating the views of the Faculty of that institution on this point:—

In their last announcement, the Faculty of Jefferson Medical College stated, that as long ago as the year 1832 they had suggested the propriety of lengthening the Medical Session; but it had never entered into their contemplation to diminish the amount of daily instruction. The objection had generally been, that the aggregate information given during a Session of Lectures in our schools is insufficient, and that, in this respect, they compare unfavorably with those of Great Britian, France and other countries of Europe. The prevalent idea, that too much is attempted to be taught in the four months generally allotted to the Medical Session, is of more recent origin.

The time usually employed in lectures during four days in the week is six hours, and it is acknowledged in all professions, and has been especially so by lawyers from Lord Coke downwards, that six hours daily ought to be devoted to professional reading. To lecture may be regarded as synonymous with 'to read'; consequently the medical student who listens to six lectures in the day may be looked upon as having been 'read to' for six hours; and there can be no essential difference between *reading* and being *read to*, except in the circumstance, that the latter is much easier for the student. The well informed and able lecturer adapts his elucidations more readily to the comprehension of his hearers than can be done in the best books. He has an opportunity of perceiving whether he is understood; and should he think he is not, he modifies or repeats his instruction. It would seem irrational to recommend to the student of law, that because he has read six hours in the day he should repeat his reading at night; and not less so to advise that this should be done by the medical student.

It is sufficient to inculcate, that he should ponder well upon the various subjects, which have been read to him during the day; and when at a loss, that he should refer to the pages of an approved text book. In so doing he need not deprive himself of proper rest and exercise, ample time for which, as well as for the enjoyment of social intercourse with the cultivated and intelligent, will be afforded, provided his time be properly apportioned to study and recreation. A part of his evenings should, in other words, be devoted to *study* not to *reading* of which he has had enough during the day. The time for farther reading will present itself when he leaves the halls of the College, to spend the interval between his College courses."

We may state, in connection with the foregoing, that the Faculty of the Medical Department of the University of Buffalo have, as yet, adopted no definitive resolution on this subject, preferring to leave it open for action until the session commences.

